# Deterministic improvements of quantum measurements with grouping of compatible operators, non-local transformations, and covariance estimates

**DOI:** 10.1038/s41534-023-00683-y

**Published:** 2023-02-22

**Authors:** Tzu-Ching Yen, Aadithya Ganeshram, Artur F. Izmaylov

**Affiliations:** 1 Chemical Physics Theory Group, Department of Chemistry, University of Toronto, Toronto, ON M5S 3H6 Canada; 2 Department of Physical and Environmental Sciences, University of Toronto, Scarborough, Toronto, ON M1C 1A4 Canada

**Keywords:** Quantum information, Method development

## Abstract

Obtaining the expectation value of an observable on a quantum computer is a crucial step in the variational quantum algorithms. For complicated observables such as molecular electronic Hamiltonians, one of the strategies is to present the observable as a linear combination of measurable fragments. The main problem of this approach is a large number of measurements required for accurate estimation of the observable’s expectation value. We consider three previously studied directions that minimize the number of measurements: (1) grouping commuting operators using the greedy approach, (2) involving non-local unitary transformations for measuring, and (3) taking advantage of compatibility of some Pauli products with several measurable groups. The last direction gives rise to a general framework that not only provides improvements over previous methods but also connects measurement grouping approaches with recent advances in techniques of shadow tomography. Following this direction, we develop two measurement schemes that achieve a severalfold reduction in the number of measurements for a set of model molecules compared to previous state-of-the-art methods.

## Introduction

Variational Quantum Algorithms (VQA) constitute one of the most promising class of applications for quantum computers in the noisy intermediate scale quantum era^1,2^. In VQAs, classically intractable optimization problems are represented as lowest eigenstates of *N*_q_-qubit operators

1 $$\begin{array}{r}\hat{H}=\mathop{\sum }\limits_{n=1}^{{N}_{{{{\rm{P}}}}}}{c}_{n}{\hat{P}}_{n},\,{\hat{P}}_{n}={\otimes }_{k = 1}^{{N}_{{{{\rm{q}}}}}}{\hat{\sigma }}_{k}\end{array}$$

where *c*_*n*_ are coefficients and ${\hat{P}}_{n}$ are tensor products of Pauli operators or identities, ${\hat{\sigma }}_{k}\in \{{\hat{x}}_{k},{\hat{y}}_{k},{\hat{z}}_{k},{\hat{1}}_{k}\}$. VQAs then solve these problems by minimizing $E({{{\boldsymbol{\theta }}}})=\left\langle \psi \left({{{\boldsymbol{\theta }}}}\right)\right\vert \hat{H}\left\vert \psi \left({{{\boldsymbol{\theta }}}}\right)\right\rangle ,$ where the quantum computer prepares the trial wavefunction $\left\vert \psi \left({{{\boldsymbol{\theta }}}}\right)\right\rangle$ and is given a task to measure *E*(***θ***), while a classical optimizer determines the optimal ***θ***. However, it was found that estimating *E*(***θ***) accurately for chemical systems requires large numbers of measurements that diminish VQA’s advantage over classical alternatives^3^.

Measuring *E*(***θ***) is indeed not a straightforward task since only *z*-Pauli operators can be measured on current digital quantum computers. A common approach to measuring the expectation value of the Hamiltonian is to present $\hat{H}$ as a sum of measurable fragments $\hat{H}={\sum }_{\alpha }{\hat{A}}_{\alpha }$. The condition for selecting ${\hat{A}}_{\alpha }$ is that they can be easily rotated into polynomial functions of *z*-Pauli operators

2 $$\begin{array}{r}{\hat{A}}_{\alpha }={\hat{U}}_{\alpha }^{{\dagger} }\left[\mathop{\sum}\limits_{i}{a}_{i{,}_{\alpha }}{\hat{z}}_{i}+\mathop{\sum}\limits_{ij}{b}_{ij,\alpha }{\hat{z}}_{i}{\hat{z}}_{j}+...\right]{\hat{U}}_{\alpha }.\end{array}$$

Then $\left\langle \psi \left({{{\boldsymbol{\theta }}}}\right)\right\vert \hat{H}\left\vert \psi \left({{{\boldsymbol{\theta }}}}\right)\right\rangle ={\sum }_{\alpha }\left\langle \psi \left({{{\boldsymbol{\theta }}}}\right)\right\vert {\hat{A}}_{\alpha }\left\vert \psi \left({{{\boldsymbol{\theta }}}}\right)\right\rangle$ where the latter can be obtained by measuring *z*-Pauli operators of ${\hat{A}}_{\alpha }$ for the rotated wavefunction ${\hat{U}}_{\alpha }\left\vert \psi \left({{{\boldsymbol{\theta }}}}\right)\right\rangle$.

Unfortunately, in general, the wavefunction $\left\vert \psi \left({{{\boldsymbol{\theta }}}}\right)\right\rangle$ is not an eigenstate of ${\hat{A}}_{\alpha }$, and thus each fragment requires a set of measurements to obtain an estimator ${\bar{A}}_{\alpha }$ for $\left\langle \psi \left({{{\boldsymbol{\theta }}}}\right)\right\vert {\hat{A}}_{\alpha }\left\vert \psi \left({{{\boldsymbol{\theta }}}}\right)\right\rangle$. The efficiency of the Hamiltonian measurement scheme is determined by the total number of measurements, *M*, needed to reach *ϵ* accuracy for *E*(***θ***). For a simple estimator of *E*(***θ***) as the sum of ${\bar{A}}_{\alpha }$ estimators, the error scales as $\epsilon =\sqrt{{\sum }_{\alpha }{{{\mbox{Var}}}}_{\psi }\left(\hat{A}_{\alpha }\right)/{m}_{\alpha }}$, where ${{{\mbox{Var}}}}_{\psi }\left(\hat{A}_{\alpha }\right)=\left\langle \psi \right\vert {\hat{A}}_{\alpha }^{2}\left\vert \psi \right\rangle -\left\langle \psi \right\vert {\hat{A}}_{\alpha }{\left\vert \psi \right\rangle }^{2}$ is the variance of each fragment, and *m*_*α*_ are the numbers of measurements allocated for each fragment, with the condition ∑_*α*_*m*_*α*_ = *M*. The optimal distribution of measurements is ${m}_{\alpha } \sim \sqrt{{{{\mbox{Var}}}}_{\psi }\left(\hat{A}_{\alpha }\right)}$, which gives the total estimator error as $\epsilon ={\sum }_{\alpha }\sqrt{{{{\mbox{Var}}}}_{\psi }\left(\hat{A}_{\alpha }\right)}/\sqrt{M}$.

This consideration shows superiority of estimators operating with a set of measurable fragments that have the lowest sum over variance square roots. For practical use of this consideration, there are two difficulties in explicit optimization of the estimator error: (1) there is an overwhelming number of choices for measurable operator fragments and (2) variance estimates require knowledge of the wavefunction. The second problem can be addressed by introducing a classically efficient proxy for the quantum wavefunction (e.g. from Hartree-Fock or configuration interaction singles and doubles (CISD) methods in quantum chemistry problems) or by utilizing the measurement results from VQAs to gain empirical estimates if classical efficient proxy cannot be found for the trial wavefunction. Yet, the search space in the first problem is so vast that it has only been addressed heuristically in previous studies. The Hamiltonian partitioning has been done in qubit space^4–11^ and in the original fermionic space with subsequent transfer of all operators into the qubit space^12,13^. An initial heuristic idea was to reduce the number of measurable fragments without accounting for variances. It was shown for several partitioning that the number of fragments is not a good proxy for the total number of measurements, and the fragments’ variances cannot be ignored^13,14^. The key element determining a particular set of measurable fragments is a class of unitary transformations ${\hat{U}}_{\alpha }$ in Eq. (2). Compared to single-qubit transformations, multi-qubit transformations are more flexible and therefore have a greater potential to minimize the total number of measurements by selecting fragments with lower variances. Yet, they also have a downside of an extra circuit overhead needed to perform the rotation before the measurement. Once the set of unitary transformations has been selected, empirically, it was found more beneficial for the estimator variance to use greedy algorithms for the Hamiltonian partitioning. In these algorithms one finds ${\hat{A}}_{\alpha }$ fragments sequentially by minimizing the norm of the difference between partial sum of ${\hat{A}}_{\alpha }$ and $\hat{H}$^13,14^. This can be rationalized considering that greedy algorithms produce first fragments with larger variances and later fragments with smaller variances. Such a distribution of variances makes sum of square roots somewhat smaller compare to the case where variances are distributed relatively equally over all fragments.

Fragmentation techniques in the qubit space are based on grouping mutually commuting Pauli products in each fragment ${\hat{A}}_{\alpha }$ [Eq. (2)]. Two types of commutativity between Pauli products are used: qubit-wise and full commutativity. The full commutativity (FC) is the regular commutativity of two operators^7^, whereas the qubit-wise commutativity (QWC) for two Pauli products is a condition when corresponding single-qubit operators commute^5^. Using either commutativity to find ${\hat{A}}_{\alpha }$, one can efficiently identify unitary operators ${\hat{U}}_{\alpha }$ from the Clifford group that bring the fragments to the form of Eq. (2) for measurement. Only one-qubit Clifford gates are sufficient for ${\hat{U}}_{\alpha }$ of the qubit-wise commuting fragments^5^, while ${\hat{U}}_{\alpha }$ for fully commuting fragments require also two-qubit Clifford gates^7^.

**Fig. 1:**
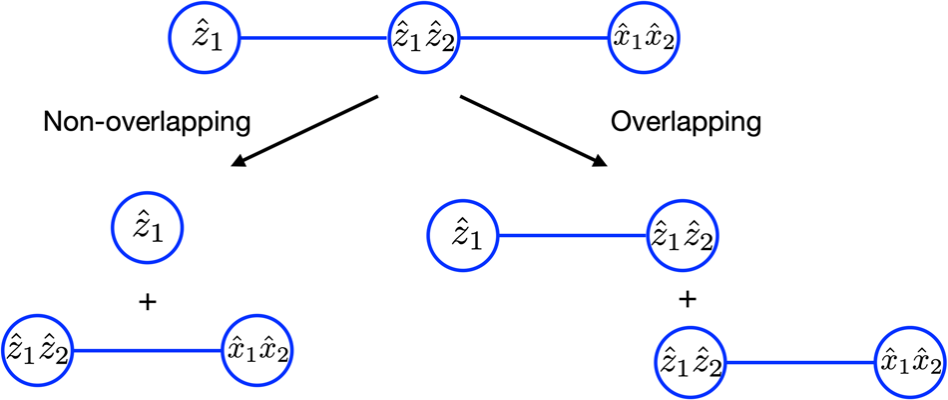
Illustration of non-overlapping and overlapping partitioning. The graph is based on full commutativity for a model Hamiltonian, $\hat{H}={c}_{1}{\hat{z}}_{1}+{c}_{2}{\hat{z}}_{1}{\hat{z}}_{2}+{c}_{3}{\hat{x}}_{1}{\hat{x}}_{2}$. Within the non-overlapping scheme the fragments are: ${\hat{A}}_{1}={c}_{1}{\hat{z}}_{1}$ and ${\hat{A}}_{2}={c}_{2}{\hat{z}}_{1}{\hat{z}}_{2}+{c}_{3}{\hat{x}}_{1}{\hat{x}}_{2}$. For the overlapping scheme based on coefficient splitting (measurement allocation) the fragments are: ${\hat{A}}_{1}={c}_{1}{\hat{z}}_{1}+{c}_{2}^{(1)}{\hat{z}}_{1}{\hat{z}}_{2}$ (${\hat{A}}_{1}={c}_{1}{\hat{z}}_{1}+{c}_{2}{\hat{z}}_{1}{\hat{z}}_{2}$) and ${\hat{A}}_{2}={c}_{2}^{(2)}{\hat{z}}_{1}{\hat{z}}_{2}+{c}_{3}{\hat{x}}_{1}{\hat{x}}_{2}$ (${\hat{A}}_{2}={c}_{2}{\hat{z}}_{1}{\hat{z}}_{2}+{c}_{3}{\hat{x}}_{1}{\hat{x}}_{2}$).

Initial QWC- and FC-based schemes had ${\hat{A}}_{\alpha }$ consisting of disjoint (non-overlapping) sets of Pauli products. Generally, each Pauli product can belong to multiple ${\hat{A}}_{\alpha }$ as long as it commutes with all terms in these fragments. This follows from non-transitivity of both FC and QWC as binary relations: if ${\hat{P}}_{1}$ commutes with ${\hat{P}}_{2}$, and ${\hat{P}}_{2}$ commutes with ${\hat{P}}_{3}$, this does not lead to commutativity of ${\hat{P}}_{1}$ and ${\hat{P}}_{3}$. For the measurement problem, ${\hat{P}}_{1}$ and ${\hat{P}}_{3}$ form separate measurable groups while ${\hat{P}}_{2}$ can be measured within both of these groups. Here, ${\hat{P}}_{2}$ constitutes an overlapping element for the ${\hat{P}}_{1}$ and ${\hat{P}}_{3}$ groups (see Fig. 1 where ${\hat{P}}_{1}$, ${\hat{P}}_{2}$, and ${\hat{P}}_{3}$ are ${\hat{z}}_{1}$, ${\hat{z}}_{1}{\hat{z}}_{2}$, and ${\hat{x}}_{1}{\hat{x}}_{2}$ respectively). Recent developments based on shadow tomography^15–18^ and grouping^19,20^ techniques exploiting overlapping fragments found considerable reduction in the number of needed measurements over non-overlapping grouping schemes. However, all non-overlapping schemes used in those comparisons did not use the greedy approach. Since within qubit-based partitioning schemes there are multiple estimator improvement techniques, it is interesting to assess them all systematically.

In this work, we assess improvements in the total number of measurements from introducing a series of ideas: (1) grouping commuting operators using the greedy approach^14^, (2) involving non-local (entangling) unitaries for measuring groups of fully commuting Pauli products^6–8,11^, and (3) taking advantage of compatibility of some Pauli products with several measurable groups (i.e. overlapping grouping)^15–20^. It is shown that these ideas, used separately or combined, can give rise to schemes superior to prior art within grouping and shadow tomography techniques^16,19^. One of the most striking findings is that using only greedy non-overlapping grouping within the QWC approach can already surpass the performance of recent techniques that employed overlapping local frames. We do not consider fermionic-algebra-based techniques here because they do not allow overlapping grouping while all other improvements were already discussed for them^13^. Other measurement techniques that do not involve grouping of Hamiltonian terms are also outside of the scope of the current work^21–25^.

## Results

We assess the performance of the proposed approaches (IMA, GMA, and ICS) in comparison to prior works (LF, SI, and classical-shadow-based algorithms) in estimating energy expectation values for ground eigen-states of several molecular electronic Hamiltonians. The qubit Hamiltonians were generated using the STO-3G basis and the BK transformation. The nuclear geometries for the Hamiltonians are R(H–H) = 1Å (H_2_), R(Li–H) = 1Å (LiH), R(Be–H) = 1Å with collinear atomic arrangement (BeH_2_), R(O–H) = 1Å with ∠*HOH* = 107. 6^∘^ (H_2_O), and R(N–H) = 1Å with ∠*HNH* = 107^∘^ (NH_3_). The overlapping groups (${{{{\mathcal{P}}}}}_{\alpha }$) of the proposed methods are obtained from an extension of the sorted insertion technique (see Supplementary Note 1). The initial measurement allocations or coefficient splittings are derived from measurement allocations of the SI technique using exact or CISD wavefunctions.

To illustrate the relative performance of our methods, Table 1 presents the Hamiltonian estimator variances based on covariances calculated with the exact wavefunction (Supplementary Note 5 illustrates the connection of these variances with energy errors). Lower variances in SI compared to those in the largest first (LF) algorithm are consistent with earlier findings^14^. All proposed methods result in lower variances than those in SI. As the most flexible approach, the coefficient splitting method ICS achieves the lowest variances. GMA has a slight edge over IMA in estimator variances, but due to the computational cost of GMA, we will only consider IMA from here on.

**Table 1:** Variances of the Hamiltonian estimators using exact wavefunction.

Systems	LF	SI	IMA	GMA	ICS
Qubit-wise commutativity					
H_2_	0.136	0.136	0.136	0.136	0.136
LiH	5.84	2.09	1.73	1.52	0.976
BeH_2_	14.3	6.34	5.60	5.26	4.29
H_2_O	116	48.6	27.9	18.8	13.5
NH_3_	352	97.0	83.3	62.1	44.8
Full commutativity					
H_2_	0.136	0.136	0.136	0.136	0.136
LiH	1.43	0.882	0.647	0.517	0.232
BeH_2_	5.18	1.11	1.02	0.974	0.459
H_2_O	43.4	7.59	5.88	4.27	1.50
NH_3_	78.7	18.8	13.6	9.35	3.32

Variances of the Hamiltonian estimators in different methods calculated with the exact wavefunction: largest first (LF), sorted insertion (SI), iterative measurement allocation (IMA), gradient-based measurement allocation (GMA), and iterative coefficient splitting (ICS). Covariances calculated with the exact wavefunction were used for finding optimal parameters in all methods.

Table 2 shows the number of optimization variables in the measurement allocation and coefficient splitting techniques. For the measurement allocation approaches (IMA and GMA) the number of variables is equal to the number of measurable groups. For the qubit-wise (full) commutativity, the number of such groups scales as ~*N*_P_/3 ($\sim {N}_{{{{\rm{q}}}}}^{3}$) since on average each group contains three (*N*_q_) Pauli products. For relatively small molecules in our set (i.e. only few atoms), *N*_P_ scales as ${N}_{{{{\rm{q}}}}}^{4}$. In the coefficient splitting approach, the number of variables is a product of *N*_P_ and an average number of measurable groups that are compatible with an average Pauli product. For our model systems, it was found empirically that the latter number grows as $\sim {N}_{{{{\rm{q}}}}}^{3}$ for the qubit-wise commutativity, whereas for the full commutativity the number is within a range of [0.4, 2.3] and thus can be considered relatively constant. These considerations clarify why the measurement allocation techniques can be employed for both commutativities, but the coefficient splitting without extra constraints can be afforded only for full commutativity.

**Table 2:** Number of optimization variables.

Systems	*N*_*q*_	*N*_*P*_	QWC		FC	
			MA	CS	MA	CS
H_2_	4	15	3	4	2	6
LiH	12	631	155	3722	42	1466
BeH_2_	14	666	183	5946	36	1203
H_2_O	14	1086	334	11192	50	1823
NH_3_	16	3609	1359	61137	122	6138

The number of optimization variables in the measurement allocation (MA) and coefficient splitting (CS) methods for the full and qubit-wise commutativities (FC and QWC) and different molecular electronic Hamiltonians. *N*_q_ is the number of qubits, and *N*_P_ is the number of Pauli products.

To compare the proposed methods to the classical shadow tomography techniques (Derand^16^ and OGM^19^), we consider qubit-wise commuting (QWC) grouping methods that do not require non-local (entangling) transformations and use approximate covariances obtained from CISD wavefunction to choose optimal parameters for the other algorithms (Table 3). Unlike the original OGM treatment, we avoid deleting measurement bases to compare all methods on an equal footing. Comparison between the non-overlapping techniques (LF and SI) and classical shadow techniques reveals that only employing the greedy approach to QWC grouping in SI is already enough to surpass the classical shadow tomography techniques. Due to sensitivity of ICS optimization to inaccurate covariance estimates, we only optimize coefficients of Pauli products with the top 90% CISD variances. The remaining Pauli products have their coefficients frozen to that of the SI scheme. In accord with results of Table 1, both IMA and ICS outperform SI even when approximate covariances are used.

**Table 3:** Variances with qubit wise commuting fragments.

Systems	LF	OGM	Derand	SI	IMA	ICS
H_2_	0.136	0.173	0.144	0.136	0.136	0.136
LiH	5.84	3.50	3.74	2.09	1.73	1.07
BeH_2_	14.3	18.3	12.5	6.34	5.60	4.54
H_2_O	116	148	114	48.6	27.9	15.9
NH_3_	352	305	251	97.0	83.4	53.8

Variances of Hamiltonian estimators with qubit wise commuting fragments: largest first (LF), overlapped grouping measurement (OGM), derandomization (Derand), sorted insertion (SI), iterative measurement allocation (IMA), and iterative coefficient splitting (ICS). The LF, SI, IMA, and ICS algorithms utilize CISD wavefunctions for choosing parameters, but the final variances are computed using exact wavefunctions.

Similarly, switching to application of CISD variances to optimize grouping based on full commutativity clearly shows several times improvements in the number of measurements for IMA and ICS compared to non-overlapping techniques (Table 4).

**Table 4:** Variances of Hamiltonian estimators with fully commuting fragments.

Systems	LF	SI	IMA	ICS
H_2_	0.136	0.136	0.136	0.136
LiH	1.43	0.882	0.647	0.295
BeH_2_	5.19	1.11	1.02	0.543
H_2_O	43.4	7.59	5.89	2.21
NH_3_	78.8	18.8	13.7	4.95

Variances of Hamiltonian estimators with fully commuting fragments: largest first (LF), sorted insertion (SI), iterative measurement allocation (IMA), and iterative coefficient splitting (ICS). All algorithms utilize CISD wavefunctions for choosing parameters, but the final variances are computed using exact wavefunctions.

To explore possible advantages of the IMA and ICS scheme in cases where approximate covariances cannot be obtained from classical wavefunction approximations, we consider a case of random wavefunctions. For all molecular systems corresponding wavefunctions were randomly generated by selecting their coefficients in computational basis from a uniform distribution and renormalizing. These wavefunctions were used to generate exact covariances needed for the overlapping grouping optimizations in IMA and ICS. Table 5 shows that using exact covariances IMA and ICS can improve the number of measurements even for randomly generated wavefunctions.

**Table 5:** Average estimator variances with random wavefunction and exact covariances.

Systems	Derand (QWC)	SI (QWC)	IMA (QWC)	ICS (QWC)	SI (FC)	IMA (FC)	ICS (FC)
H_2_	0.241	0.233	0.226	0.219	0.202	0.185	0.177
LiH	13.6	11.2	8.64	8.59	7.43	6.18	6.13
BeH_2_	45.5	38.7	29.3	29.2	24.0	21.2	21.1
H_2_O	799	715	517	505	478	410	406
NH_3_	865	657	392	391	324	249	246

Average variances of the Hamiltonian estimators in methods using qubit-wise and full commutativity (QWC and FC) calculated from 4 random wavefunctions for each system. Optimal parameters for sorted insertion (SI), iterative measurement allocation (IMA), iterative coefficient splitting (ICS) are obtained using the exact covariances.

For considering a more realistic scenario where covariances cannot be evaluated because the wavefunction is not known, it was assumed that covariances can be obtained through accumulated measurement results for any trial wavefunction. A modest 1000 measurements were considered for each fragment to estimate covariances between simultaneously measured Pauli products: ${\hat{P}}_{i}$, ${\hat{P}}_{j}$. We simulated such measurements to obtain approximate $\left\langle {\hat{P}}_{i}\right\rangle$, $\left\langle \hat{P}_{j}\right\rangle$ and $\left\langle {\hat{P}}_{i}{\hat{P}}_{j}\right\rangle$. If a Pauli product appears in multiple fragments, measurements in all fragments contribute to the expectation value estimate. The approximate expectation values allow us to estimate covariances between ${\hat{P}}_{i}$ and ${\hat{P}}_{j}$, which are then used to obtain results shown in Table 6. The results reaffirm that IMA and ICS are the most efficient measurement methods among the presented even with approximate covariances. Note that incorporating measured covariances into measurement optimization can be done more efficiently, as detailed in ref. ^20^.

**Table 6:** Average number of measurements with random wavefunction and approximate covariances.

Systems	Derand (QWC)	SI (QWC)	IMA (QWC)	ICS (QWC)	SI (FC)	IMA (FC)	ICS (FC)
H_2_	0.241	0.236	0.230	0.222	0.204	0.187	0.179
LiH	13.6	11.4	8.80	8.81	7.47	6.22	6.23
BeH_2_	45.5	38.9	29.5	29.7	24.0	21.2	21.3
H_2_O	799	715	517	510	480	410	407
NH_3_	865	658	394	393	324	249	249

Average number of measurements in millions that are required to have *ϵ* = 10^−3^ a.u. accuracy in the true expectation values of 4 random wavefunctions for each system. Note that due to the choice of *ϵ* and use of millions as units, the numbers here are similar to those in Table 5. These numbers include measurements used for estimating covariances for sorted insertion (SI), iterative measurement allocation (IMA), and iterative coefficient splitting (ICS). The obtained approximate covariances were employed to determine optimal parameters.

Interestingly, the advantage of ICS over IMA diminishes when we use random wavefunctions. This suggests that the extra degrees of freedom in optimizing ${c}_{k}^{(\alpha )}$ is not more beneficial to reducing estimator variance than the simple choice ${c}_{k}^{(\alpha )}={c}_{k}{m}_{\alpha }/M$. Indeed, in the case of random wavefunctions, any Pauli product ${\hat{P}}_{k}$ tends to not correlate with fragments consisting of many Pauli products, whose covariances with ${\hat{P}}_{k}$ are distributed symmetrically about zero. In such case, it makes intuitive sense to choose ${c}_{k}^{(\alpha )}$ to be proportional to the number of times that ${\hat{P}}_{k}$ is measured in each group.

## Discussion

We assessed multiple ideas for reduction of the number of measurements required to accurately obtain the expectation value of any operator that can be written as a sum of Pauli products. Since these ideas can be used separately or combined, our main goal was to understand the impact on the number of measurements and incurred computational cost of each idea. Exploring the idea of Pauli products’ compatibility led to the realization that the coefficient splitting framework is the most general implementation of this idea for the grouping methods.

Among previously suggested measurement allocation approaches^15,16,19,20^ only ref. ^20^ went beyond QWC fragments and utilized their FC counterparts for the first time. In addition, in ref. ^20^ analytical formulas for the measurement error were derived and the measurement shots were distributed according to the knowledge on the covariances. Although these techniques have shown performance superior to that of the non-overlapping measurement scheme based on graph-coloring algorithms, by employing a greedy heuristic the non-overlapping scheme can already outperform the Derand and OGM techniques. Thus, for future developments, new approaches need to be compared with greedy grouping-based algorithms rather than with grouping approaches that try to minimize the overall number of measurable groups (e.g. LF).

Unlike previous classical shadow techniques that focus on qubit-wise commuting groups, we also considered measuring techniques involving non-local (entangling) transformations that allow one to measure groups of fully commuting Pauli products. An efficient implementation of these non-local transformations using Clifford gates was proposed by Gottesman^26^ and would introduce only $O({N}_{{{{\rm{q}}}}}^{2}/\log {N}_{{{{\rm{q}}}}})$ CNOT gates. The schemes based on fully commuting groups outperform their qubit-wise commuting counterparts up to a factor of seven in variances of the expectation value estimators. Even accounting for increase of the number of measurements related to uncertainties from a lower fidelity of CNOT gates, fully commuting grouping schemes require fewer numbers of measurements than their qubit-wise commuting counterparts^27^.

Taking advantage of compatibility of some Pauli products with members of multiple measurable groups (i.e. overlapping groups idea) can be generally presented as augmenting the measurable groups with all Pauli products compatible with initial members of these groups. Then the coefficients of Pauli products entering multiple groups are optimized to lower the estimator variance, with the constraint that the sum over coefficients in different groups for each Pauli product is equal to the coefficient of the Pauli product in the Hamiltonian. This coefficient splitting approach incorporates as a special case a heuristic technique of optimizing measurement allocation for overlapping measurable groups.

Even though the coefficient splitting variance minimization provides the lowest variances among all studied approaches, it requires optimizing a large number of variables: $\sim {N}_{{{{\rm{q}}}}}^{4}$ ($\sim {N}_{{{{\rm{q}}}}}^{7}$) for full (qubit-wise) commutativity. Due to certain restrictions, the measurement allocation approach is much more economical in the number of optimization variables: $\sim {N}_{{{{\rm{q}}}}}^{3}$ ($\sim {N}_{{{{\rm{q}}}}}^{4}$) for full (qubit-wise) commutativity. Another contributor of the computational cost of these techniques is calculation of the variance gradients. To reduce the computational cost of this part we proposed iterative schemes, the ICS method converges to true extrema, while the IMA scheme deviates from extrema. IMA and ICS provide up to forty and eighty percent reduction in the number of measurements required compared to corresponding best non-overlapping techniques.

Both IMA and ICS use approximate covariances between Pauli products to lower the estimator variance. Use of CISD wavefunction for obtaining these covariances for physically relevant states generally show improvements comparable to those obtained using the exact covariances. In cases where classically efficient approximate wavefunctions are not available, approximate covariances can be obtained via quantum measurements.

## Methods

### Estimator for non-overlapping Pauli groups

All measurable fragments ${\hat{A}}_{\alpha }$ are linear combinations of mutually commuting or qubit-wise commuting Pauli products

3 $$\begin{array}{r}{\hat{A}}_{\alpha }=\mathop{\sum}\limits_{k}{c}_{k}{\hat{P}}_{k},\,{\hat{P}}_{k}\in {{{{\mathcal{P}}}}}_{\alpha },\end{array}$$

where ${{{{\mathcal{P}}}}}_{\alpha }$ are disjoint sets of Pauli products measured as parts of corresponding ${\hat{A}}_{\alpha }$, and *c*_*k*_ are coefficients of ${\hat{P}}_{k}$ in the Hamiltonian. The commutativity between Pauli products within ${{{{\mathcal{P}}}}}_{\alpha }$ implies a common eigen-basis **B**_*α*_, where one can measure all the members of ${{{{\mathcal{P}}}}}_{\alpha }$. Initial proposals to find these fragments aim to minimize the total number of fragments using graph coloring algorithms, such as the largest first (LF) algorithm^5,7^. But later the sorted insertion (SI) algorithm employing the greedy approach was found to produce better groups in terms of the number of measurements^14^.

Let $\bar{H}$ denotes the estimator for $\left\langle \psi \right\vert \hat{H}\left\vert \psi \right\rangle$; it is a sum of estimators for its parts

4 $$\begin{array}{r}\bar{H}=\mathop{\sum }\limits_{\alpha =1}^{L}{\bar{A}}_{\alpha }.\end{array}$$

Each ${\bar{A}}_{\alpha }$ comes from *m*_*α*_ repeated measurements of ${\hat{A}}_{\alpha }$,

5 $$\begin{array}{r}{\bar{A}}_{\alpha }=\frac{1}{{m}_{\alpha }}\mathop{\sum }\limits_{i=1}^{{m}_{\alpha }}{A}_{\alpha ,i},\end{array}$$

where *A*_*α*,*i*_ is the *i*-th measurement result of ${\hat{A}}_{\alpha }$. The variance of $\bar{H}$ is

6 $$\begin{array}{r}\,{{\mbox{Var}}}\,\left(\bar{H}\right)=\mathop{\sum }\limits_{\alpha =1}^{L}\,{{\mbox{Var}}}\,\left(\bar{A}_{\alpha }\right),\end{array}$$

where $\,{{\mbox{Var}}}\,\left(\bar{A}_{\alpha }\right)$ are variances of estimators characterizing differences between ${\bar{A}}_{\alpha }$ and the true expectation values $\left\langle \psi \right\vert {\hat{A}}_{\alpha }\left\vert \psi \right\rangle$. Note that covariances between different fragments ${{{\rm{Cov}}}}({\bar{A}}_{\alpha },{\bar{A}}_{\beta })$ are zero because measurements of different fragments are done independently. $\,{{\mbox{Var}}}\,\left(\bar{A}_{\alpha }\right)$ can be evaluated using quantum operator variances ${{{\mbox{Var}}}}_{\psi }\left(\hat{A}_{\alpha }\right)$, $\,{{\mbox{Var}}}\,\left(\bar{A}_{\alpha }\right)={{{\mbox{Var}}}}_{\psi }\left(\hat{A}_{\alpha }\right)/{m}_{\alpha }$, which leads to the Hamiltonian estimator variance as

7 $$\begin{array}{r}\,{{\mbox{Var}}}\,\left(\bar{H}\right)=\mathop{\sum }\limits_{\alpha =1}^{L}\frac{1}{{m}_{\alpha }}{{{\mbox{Var}}}}_{\psi }\left(\hat{A}_{\alpha }\right).\end{array}$$

Using the constraint *M* = ∑_*α*_*m*_*α*_ one can minimize $\,{{\mbox{Var}}}\,\left(\bar{H}\right)$ with respect to *m*_*α*_^14,28^ which gives

8 $$\begin{array}{r}\,{{\mbox{Var}}}\,{\left(\bar{H}\right)}_{\min }=\frac{1}{M}{\left(\mathop{\sum}\limits_{\alpha }\sqrt{{{{\mbox{Var}}}}_{\psi }\left({\hat{A}}_{\alpha }\right)}\right)}^{2}.\end{array}$$

with

9 $$\begin{array}{r}{m}_{\alpha }^{{{{\rm{(min)}}}}}=\sqrt{{{{\mbox{Var}}}}_{\psi }\left({\hat{A}}_{\alpha }\right)}\frac{{\sum }_{\beta }\sqrt{{{{\mbox{Var}}}}_{\psi }\left({\hat{A}}_{\beta }\right)}}{\,{{\mbox{Var}}}\,\left(\bar{H}\right)}\end{array}$$

Note that this minimization gives generally non-integer ${m}_{\alpha }^{{{{\rm{(min)}}}}}$. Here and in what follows we will always assume taking the integer approximation ⌊*m*_*α*_⌋ for obtained *m*_*α*_ if *m*_*α*_ are used as integer quantities. In case of large *M*, the difference between *m*_*α*_ and ⌊*m*_*α*_⌋ in the estimator variance is negligible.

The minimum variance in Eq. (8) is generally lower if there is an uneven distribution of ${{{\mbox{Var}}}}_{\psi }\left({\hat{A}}_{\alpha }\right)$. This motivates the sorted insertion (SI) algorithm to employ the greedy approach to achieve an uneven distribution of norms of coefficients in fragments, which was found to produce the lowest variances for the energy estimators out of all non-overlapping grouping techniques^14^.

In practice, quantum variances ${{{\mbox{Var}}}}_{\psi }\left(\hat{A}_{\alpha }\right)$ are not known a priori. They can be evaluated using covariances between Pauli products,

10 $$\begin{array}{r}{{{\mbox{Var}}}}_{\psi }\left({\hat{A}}_{\alpha }\right)=\mathop{\sum}\limits_{jk}{c}_{j}{c}_{k}{{{\mbox{Cov}}}}_{\psi }\left({\hat{P}_{j},{\hat{P}}_{k}}\right)\end{array}$$

11 $$\begin{array}{l}{{{\mbox{Cov}}}}_{\psi }\left({\hat{P}_{j},{\hat{P}}_{k}}\right)=\left\langle \psi \right\vert {\hat{P}}_{j}{\hat{P}}_{k}\left\vert \psi \right\rangle -\left\langle \psi \right\vert {\hat{P}}_{j}\left\vert \psi \right\rangle \\ \qquad\qquad\qquad\qquad\times \left\langle \psi \right\vert {\hat{P}}_{k}\left\vert \psi \right\rangle ,\end{array}$$

where ${\hat{P}}_{j},\,{\hat{P}}_{k}\in {{{{\mathcal{P}}}}}_{\alpha }$. The covariances for different Pauli products are generally non-zero because all of these Pauli products are measured together within the same fragment. The covariances can be approximated for molecular Hamiltonians using approximate wavefunctions obtained on a classical computer. Configuration interaction singles and doubles (CISD) is one example for obtaining approximation for $\left\vert \psi \right\rangle$ that will be used in the current work. Alternatively, the measurements results obtained from measurement basis **B**_*α*_ can help estimate the covariances between Pauli products of ${{{{\mathcal{P}}}}}_{\alpha }$ during VQA cycles.

### Optimization by coefficient splitting

Many Pauli products in the Hamiltonian can be measured in multiple fragments because of their compatibility with other members of those fragments. The coefficient splitting approach, briefly mentioned in ref. ^14^, takes advantage of this opportunity by splitting coefficients of Pauli products that are compatible with multiple fragments

12 $$\begin{array}{r}{\hat{A}}_{\alpha }=\mathop{\sum}\limits_{k}{c}_{k}^{(\alpha )}{\hat{P}}_{k},\,{\hat{P}}_{k}\in {{{{\mathcal{P}}}}}_{\alpha }\end{array}$$

13 $$\begin{array}{r}{c}_{k}=\mathop{\sum}\limits_{\alpha \in {{{{\mathcal{I}}}}}_{k}}{c}_{k}^{(\alpha )}\end{array}$$

where ${{{{\mathcal{I}}}}}_{k}$ is a set of group indices *α* corresponding to fragments ${\hat{A}}_{\alpha }$ whose members are compatible with ${\hat{P}}_{k}$ (see Fig. 1 for an example). To find fragments ${\hat{A}}_{\alpha }$ and to establish compatibility relations between their members we developed an extension of the SI algorithm detailed in Supplementary Note 1. The SI algorithm was taken as the basis of this extension because it produces fragments with a lowest estimator variance among all non-overlapping grouping techniques. From here on, we assume use of the extension for methods proposed in this work.

Note that the equations for the estimator variance and the optimal measurement distribution remain the same [Eqs. (9) and (8)]. However, freedom in the coefficient splitting approach [Eq. (13)] can be used to minimize the Hamiltonian estimator variance [Eq. (8)].

A straightforward approach to minimization of $\,{{\mbox{Var}}}\,\left(\bar{H}\right)$ with respect to ${c}_{k}^{(\alpha )}$ is to use analytical gradients $\partial \,{{\mbox{Var}}}\,\left(\bar{H}\right)/\partial {c}_{k}^{(\alpha )}$. The gradients are non-linear functions of ${c}_{k}^{(\alpha )}$ and computing them becomes computationally expensive as the number of ${c}_{k}^{(\alpha )}$ grows with the size of the system. As a computationally more efficient alternative, we propose an iterative heuristic that quickly converges to a zero gradient solution.

#### Iterative coefficient splitting (ICS)

Given a particular choice of ${c}_{k}^{(\alpha )}$ and its optimal *m*_*α*_, the procedure consists of iteratively applying two steps: (1) optimizing ${c}_{k}^{(\alpha )}$ with fixed *m*_*α*_ and (2) updating *m*_*α*_ for evaluated ${c}_{k}^{(\alpha )}$ using Eq. (9). For step 1, we solve a linear system of equations originating from the $\frac{\partial \,{{\mbox{Var}}}\,\left(\bar{H}\right)}{\partial {c}_{k}^{(\alpha )}}=0$ condition (see Supplementary Note 2 for details).

If the number of ${c}_{k}^{(\alpha )}$ overcomes computationally affordable limits, one can always limit the minimization to a selected subset of ${c}_{k}^{(\alpha )}$. The criteria for the suitable subset can be the ${\hat{P}}_{k}$ variances, which correlate with magnitudes of their covariances and therefore the importance of their coefficients for $\,{{\mbox{Var}}}\,\left(\bar{H}\right)$.

### Optimization by measurement allocation

Another approach to reducing the Hamiltonian estimator variance is to measure each Pauli product as a member of as many compatible measurable fragments as possible. This idea was used in classical shadow tomography methods based on local transformations for measurement of Pauli products^15,16^ and grouping techniques for qubit-wise commuting^19^ and fully commuting^20^ fragments. First, for a particular Pauli product ${\hat{P}}_{k}$, one finds a set of measurement bases **B**_*α*_ where ${\hat{P}}_{k}$ can be measured (see Fig. 1 for an example, by a measurement group this method considers a set of compatible Pauli products). Then, all measurement results for ${\hat{P}}_{k}$ obtained in **B**_*α*_ are used to estimate ${\bar{P}}_{k}$:

14 $$\begin{array}{r}{\bar{P}}_{k}=\frac{1}{{M}_{k}}\mathop{\sum}\limits_{\alpha \in {{{{\mathcal{I}}}}}_{k}}\mathop{\sum }\limits_{i=1}^{{m}_{\alpha }}{P}_{k,i}^{(\alpha )},\end{array}$$

where ${P}_{k,i}^{(\alpha )}$ is the *i*-th measurement result of ${\hat{P}}_{k}$ measured in basis **B**_*α*_, and ${M}_{k}={\sum }_{\alpha \in {{{{\mathcal{I}}}}}_{k}}{m}_{\alpha }$ is the total number of times ${\hat{P}}_{k}$ is measured. ${\bar{P}}_{k}$ are used in the Hamiltonian estimator as $\bar{H}={\sum }_{k}{c}_{k}{\bar{P}}_{k}$. The variance of $\bar{H}$ is

15 $$\begin{array}{r}\,{{\mbox{Var}}}\,\left(\bar{H}\right)=\mathop{\sum}\limits_{jk}{c}_{j}{c}_{k}\,{{\mbox{Cov}}}\,\left({\bar{P}_{j},{\bar{P}}_{k}}\right)\end{array}$$

16 $$\begin{array}{r}= \mathop{\sum}\limits_{jk}\frac{{c}_{j}{c}_{k}}{{M}_{j}{M}_{k}}\mathop{\sum}\limits_{\begin{array}{c}\scriptstyle{\alpha \in {{{{\mathcal{I}}}}}_{j}},\\ \scriptstyle{\beta \in {{{{\mathcal{I}}}}}_{k}}\end{array}}\mathop{\sum }\limits_{u=1}^{{m}_{\alpha }}\mathop{\sum }\limits_{v=1}^{{m}_{\beta }}\,{{\mbox{Cov}}}\,\left({P}_{j,u}^{(\alpha )},{P}_{k,v}^{(\beta )}\right)\end{array}$$

To proceed further, it is important to distinguish covariances between Pauli products measured within the same fragment and in different fragments. The former correspond to *α* = *β* and *u* = *v* in Eq. (16) and generally are non-zero, while the latter (*α* ≠ *β* or *u* ≠ *v*) are zero

17 $$\begin{array}{l}\,{{\mbox{Var}}}\,\left({\bar{H}}\right)=\mathop{\sum}\limits_{jk}\frac{{c}_{j}{c}_{k}}{{M}_{j}{M}_{k}}\mathop{\sum}\limits_{\begin{array}{c}\scriptstyle{\alpha \in {{{{\mathcal{I}}}}}_{j}},\\ \scriptstyle{\beta \in {{{{\mathcal{I}}}}}_{k}}\end{array}}\mathop{\sum }\limits_{u=1}^{{m}_{\alpha }}\mathop{\sum }\limits_{v=1}^{{m}_{\beta }}{\delta }_{\alpha \beta }{\delta }_{uv}{{{\mbox{Cov}}}}_{\psi }\left({\hat{{P}}_{j},{\hat{P}}_{k}}\right)\\ \qquad\qquad\;=\mathop{\sum}\limits_{jk}\frac{{c}_{j}{c}_{k}}{{M}_{j}{M}_{k}}\mathop{\sum}\limits_{\alpha \in {{{{\mathcal{I}}}}}_{j}\cap {{{{\mathcal{I}}}}}_{k}}{m}_{\alpha }{{{\mbox{Cov}}}}_{\psi }\left({\hat{{P}}_{j},{\hat{P}}_{k}}\right).\end{array}$$

Note that the key element in deriving this Hamiltonian estimator variance is the consideration that if a Pauli product is measured as a part of a certain group, all members of this group contribute to the average and to the variance. Thus, the variance of each group gives rise to covariances between its members. Since the covariances in different groups are different in magnitude, placing a particular Pauli product in all compatible groups can be sub-optimal for the total variance of the Hamiltonian estimator (an example illustrating this phenomenon is given in Supplementary Note 4).

Dependencies of *M*_*j*_ and *M*_*k*_ on *m*_*α*_ in $\,{{\mbox{Var}}}\,\left(\bar{H}\right)$ [Eq. (17)] make finding the optimal measurement allocation in the analytic form infeasible. To minimize $\,{{\mbox{Var}}}\,\left(\bar{H}\right)$ with respect to *m*_*α*_ in Eq. (17) one can numerically optimize *m*_*α*_ as positive variables with restriction ∑_*α*_*m*_*α*_ = *M*. We will refer to this strategy as the measurement allocation approach.

Interestingly, the measurement allocation technique is equivalent to a restricted coefficient splitting optimization with ${c}_{k}^{(\alpha )}={c}_{k}{m}_{\alpha }/{M}_{k}$. Indeed, substituting ${c}_{k}^{(\alpha )}$ for *m*_*α*_ in ${\hat{A}}_{\alpha }$ and using Eq. (7), we obtain $\,{{\mbox{Var}}}\,\left(\bar{H}\right)$ as

18 $$\begin{array}{l}\,{{\mbox{Var}}}\,\left({\bar{H}}\right)=\mathop{\sum}\limits_{\alpha }\frac{1}{{m}_{\alpha }}\mathop{\sum}\limits_{jk:\alpha \in {{{{\mathcal{I}}}}}_{j}\cap {{{{\mathcal{I}}}}}_{k}}{{{\mbox{Cov}}}}_{\psi }\left(\frac{{m}_{\alpha }}{{M}_{j}}{c}_{j}{\hat{P}}_{j},\frac{{m}_{\alpha }}{{M}_{k}}{c}_{k}{\hat{P}}_{k}\right)\\ \qquad \qquad\,=\mathop{\sum}\limits_{jk}\frac{{c}_{j}{c}_{k}}{{M}_{j}{M}_{k}}\mathop{\sum}\limits_{\alpha \in {{{{\mathcal{I}}}}}_{j}\cap {{{{\mathcal{I}}}}}_{k}}{m}_{\alpha }{{{\mbox{Cov}}}}_{\psi }\left({\hat{{P}}_{j},{\hat{P}}_{k}}\right),\end{array}$$

which agrees with Eq. (17).

One can formulate approximation for gradients of $\,{{\mbox{Var}}}\,\left(\bar{H}\right)$ with respect to continuous proxy of *m*_*α*_ (see Supplementary Note 3), which leads to a gradient descent scheme that we will refer to as gradient-based measurement allocation (GMA). Yet, a computationally more efficient, non-gradient iterative scheme was found and detailed below.

#### Iterative measurement allocation (IMA)

Given an initial guess for ${m}_{\alpha }^{(0)}$ and resulting ${M}_{k}^{(0)}$, the corresponding coefficient splitting partitioning of the Hamiltonian is

19 $$\begin{array}{r}\hat{H}=\mathop{\sum }\limits_{\alpha }^{L}{\hat{A}}_{\alpha }^{(0)},\end{array}$$

where

20 $$\begin{array}{r}{\hat{A}}_{\alpha }^{(0)}=\mathop{\sum}\limits_{k}\frac{{m}_{\alpha }^{(0)}}{{M}_{k}^{(0)}}{c}_{k}{\hat{P}}_{k},\,{\hat{P}}_{k}\in {{{{\mathcal{P}}}}}_{\alpha }.\end{array}$$

Recall that the optimal measurement allocation for any coefficient splitting is given by Eq. (9). Thus, we use this optimal allocation to update ${m}_{\alpha }^{(i)}$ as

21 $$\begin{array}{r}{m}_{\alpha }^{(i)}\to {m}_{\alpha }^{(i+1)}\propto \sqrt{{{{\mbox{Var}}}}_{\psi }\left({\hat{A}}_{\alpha }^{(i)}\right)},\end{array}$$

which leads to the update in measurable groups

22 $$\begin{array}{r}{\hat{A}}_{\alpha }^{(i)}\to {\hat{A}}_{\alpha }^{(i+1)}=\mathop{\sum}\limits_{k}\frac{{m}_{\alpha }^{(i+1)}}{{M}_{k}^{(i+1)}}{c}_{k}{\hat{P}}_{k},\,{\hat{P}}_{k}\in {{{{\mathcal{P}}}}}_{\alpha }\end{array}$$

Since there is no guarantee that each iteration will necessarily lower $\,{{\mbox{Var}}}\,\left(\bar{H}\right)$ in Eq. (17), we repeat these steps multiple times and choose *m*_*α*_ that result in the lowest estimator variance. Empirically, the procedure finds the best measurement allocation in first few cycles.

### Method summary

Conceptually, there are three approaches described above: non-overlapping grouping, coefficient splitting, and measurement allocation. For all of them expectation value of the Hamiltonian is a sum of estimators for expectation values of fragments $\bar{H}={\sum }_{\alpha }{\bar{H}}_{\alpha }$, and the variance for the $\bar{H}$ estimator is given by Eq. (7). The differences between three methods are in the fragment definitions: non-overlapping grouping use fragments with original Hamiltonian coefficients *c*_*k*_ for Pauli products and each Pauli products entering only a single fragment, coefficient splitting and measurement allocation allow Pauli products to enter multiple groups with coefficients defined by the optimization procedure for Eq. (12) and ${c}_{k}^{(\alpha )}={c}_{k}{m}_{\alpha }/{M}_{k}$ (cf. Eq. (22)), respectively. Variables that are optimized to obtain the lowest estimator variance are the numbers of measurements *m*_*α*_ for measurement allocation and ${c}_{k}^{(\alpha )}$ and *m*_*α*_ for coefficient splitting. The main advantage of the measurement allocation approach is a much lower number of optimization variables (*m*_*α*_) compared to that of the coefficient splitting scheme (${c}_{k}^{(\alpha )}$). Yet, note that positivity of *m*_*α*_ imposes not only a limitation of measurement allocation with respect to coefficient splitting but also with respect to non-overlapping grouping. In non-overlapping grouping, ${c}_{k}^{(\alpha )}$ are either 0 or *c*_*k*_, but in measurement allocation, ${c}_{k}^{(\alpha )}$ cannot be zero.

### Supplementary information

Deterministic improvements of quantum measurements with grouping of compatible operators, non-local transformations, and covariance estimates

## Data Availability

The data that support the findings of this study are available on https://github.com/tymcr/VQE_Hamiltonian.

## Code availability

Some part of the code that supports the findings of this study is available in the OpenFermion^29^ and PySCF^30^ libraries. The implementation of ICS is available at Tequila^31^ (https://github.com/tequilahub/tequila). The rest of the code is available from the corresponding author upon request.
