# Supplementary material for: Deterministic improvements of quantum measurements with grouping of compatible operators, non-local transformations, and covariance estimates
Source: npj Quantum Inf. 2023 Feb 22;9(1):14. doi: 10.1038/s41534-023-00683-y (PMC11041696; doi:10.1038/s41534-023-00683-y)

Supplementary Material — Deterministic improvements of quantum measurements with grouping of compatible operators, non-local transformations, and covariance estimates

Tzu-Ching Yen,[1] Aadithya Ganeshram,[1] and Artur F. Izmaylov[1,2]

[1] *Chemical Physics Theory Group, Department of Chemistry, University of Toronto, Toronto, Ontario M5S 3H6, Canada*

[2] *Department of Physical and Environmental Sciences, University of Toronto Scarborough, Toronto, Ontario M1C 1A4, Canada*

SUPPLEMENTARY NOTE 1. EXTENSIONS OF SORTED INSERTION

Sorted Insertion (SI)[1] is one of the most efficient measurement schemes that utilizes non-overlapping Pauli groups. Here, we briefly review the original implementation and introduce modifications to find overlapping groups for the coefficient splitting and measurement allocation approaches.

SI partitions all the Pauli products $\mathcal{H} = \{\hat{P}_k\}$ in $\hat{H}$ into a set of non-overlapping groups $\mathbf{G} = \{\mathcal{P}_\alpha\}$ such that

$$\hat{H} = \sum_k c_k \hat{P}_k = \sum_\alpha \hat{A}_\alpha \tag{1}$$

$$\hat{A}_\alpha = \sum_{\hat{P}_k^{(\alpha)} \in \mathcal{P}_\alpha} c_k \hat{P}_k^{(\alpha)}. \tag{2}$$

SI initiates $\mathbf{G} = \emptyset$, $\alpha = 1$ and finds the partitioning through the following steps:

1. Sort Pauli products in $\mathcal{H}$ in the descending order of the magnitudes of their coefficients.

2. Examine each product $\hat{P} \in \mathcal{H}$. If $\hat{P}$ commutes with all products in $\mathcal{P}_\alpha$,

$$\mathcal{P}_\alpha \to \mathcal{P}_\alpha \cup \{\hat{P}\} \tag{3}$$

$$\mathcal{H} \to \mathcal{H} \setminus \{\hat{P}\}. \tag{4}$$

3. Add $\mathcal{P}_\alpha$ to $\mathbf{G}$. Set $\alpha \to \alpha + 1$ and repeat from step 2 until $\mathcal{H}$ is empty.

In order to obtain overlapping groups, we maintain set $\mathcal{P}_*$ to track Pauli products that are already part of some fragments, and examine whether they are compatible with the group $\mathcal{P}_\alpha$ built in step 2. Note that the order in which the Pauli products are added to the groups matters, since the additional Pauli products that are compatible with the SI groups may not be compatible between themselves. We initiate $\mathcal{P}_* = \emptyset$ and add an extra procedure between steps 2 and 3:

- For $\hat{P} \in \mathcal{P}_*$ in the order they were added to $\mathcal{P}_*$, add $\hat{P}$ to $\mathcal{P}_\alpha$ if $\hat{P}$ is compatible with all members of $\mathcal{P}_\alpha$. Then, $\forall \hat{P} \in \mathcal{P}_\alpha \setminus \mathcal{P}_*$, in the order added to $\mathcal{P}_\alpha$, set $\mathcal{P}_* \to \mathcal{P}_* \cup \{\hat{P}\}$

SUPPLEMENTARY NOTE 2. SOLVING FOR OPTIMAL COEFFICIENT SPLITTING THROUGH LINEAR SYSTEM OF EQUATION

The equation for variance of energy estimator is

$$\mathrm{Var}\left(\bar{H}\right) = \sum_{\alpha} \frac{1}{m_\alpha} \sum_{jk:\alpha\in\mathcal{I}_j\cap\mathcal{I}_k} c_j^{(\alpha)} c_k^{(\alpha)} \mathrm{Cov}_\psi\left(\hat{P}_j, \hat{P}_k\right).$$

Note that when m_α are fixed, the derivatives of $\mathrm{Var}\left(\bar{H}\right)$ with respect to $c_k^{(\alpha)}$ are linear in $c_k^{(\alpha)}$:

$$\frac{\partial \mathrm{Var}\left(\bar{H}\right)}{\partial c_k^{(\alpha)}} = \frac{2\sum_{j:\alpha\in\mathcal{I}_j} c_j^{(\alpha)} \mathrm{Cov}_\psi\left(\hat{P}_k, \hat{P}_j\right)}{m_\alpha}. \tag{5}$$

To account for the constraints in Eq. (13) in the main text, for each splitting of c_k, we fix one of the $\{c_k^{(\alpha)}\}_{\alpha\in\mathcal{I}_k}$ as $c_k^{(*_k)} = c_k - \sum_{\alpha\in\mathcal{I}_k\setminus\{*_k\}} c_k^{(\alpha)}$. The gradients become

$$\begin{aligned}\frac{\partial \mathrm{Var}\left(\bar{H}\right)}{\partial c_k^{(\alpha)}} = &\frac{2\sum_{j:\alpha\in\mathcal{I}_j} c_j^{(\alpha)} \mathrm{Cov}_\psi\left(\hat{P}_k, \hat{P}_j\right)}{m_\alpha} \\ &- \frac{2\sum_{j:*_k\in\mathcal{I}_j} c_j^{(*_k)} \mathrm{Cov}_\psi\left(\hat{P}_k, \hat{P}_j\right)}{m_{*_k}}.\end{aligned} \tag{6}$$

This allows us to find an optimal $c_k^{(\alpha)}$ by solving the linear system of equations obtained by equating the gradients to zero.

SUPPLEMENTARY NOTE 3. GRADIENTS OF MEASUREMENT ALLOCATION OPTIMIZATION

The variance of the estimator for $\langle\hat{H}\rangle$ as a function of m_α and M_k is

$$\mathrm{Var}\left(\bar{H}\right) = \sum_{ij} \frac{c_i c_j}{M_i M_j} \sum_{\alpha\in\mathcal{I}_i\cap\mathcal{I}_j} m_\alpha \mathrm{Cov}_\psi\left(\hat{P}_i, \hat{P}_j\right) \tag{7}$$

$$\begin{aligned}&= \sum_k \frac{c_k^2}{M_k} \mathrm{Var}\left(\hat{P}_k\right) \\ &+ \sum_{i>j} \sum_{\alpha\in\mathcal{I}_i\cap\mathcal{I}_j} \frac{2m_\alpha}{M_i M_j} c_i c_j \mathrm{Cov}_\psi\left(\hat{P}_i, \hat{P}_j\right).\end{aligned} \tag{8}$$

Note that M_i depend on m_α. To use gradient minimization for $\mathrm{Var}\left(\bar{H}\right)$ we substitute m_α by their continuous counterparts m'_α with conditions $m_\alpha = \lfloor M m'_\alpha \rfloor$, $m'_\alpha > 0$, and $\sum_\alpha m'_\alpha = 1$. For large enough M, this substitution allows us to approximate $\mathrm{Var}\left(\bar{H}\right)$ as

$$\mathrm{Var}\left(\bar{H}\right) \approx \frac{1}{M}\sum_\alpha \frac{1}{m'_\alpha}\mathrm{Var}\left(\bar{H}_\alpha\right). \tag{9}$$

The variance derivatives with respect to m'_α are

$$\begin{aligned}
\frac{\partial \mathrm{Var}\left(\bar{H}\right)}{\partial m'_\alpha} &= \sum_{k:\alpha\in\mathcal{I}_k} -\frac{c_k^2}{M_k^2}\mathrm{Var}\left(\hat{P}_k\right) \\
&+ \sum_{\substack{i>j:\\ \alpha\in\mathcal{I}_i\cap\mathcal{I}_j}} \left(\frac{1}{M_i M_j} - \frac{m'_\alpha}{\left(M_i\right)^2 M_j} - \frac{m'_\alpha}{M_i\left(M_j\right)^2}\right) \\
&\times 2c_i c_j \mathrm{Cov}_\psi\left(\hat{P}_i, \hat{P}_j\right) \\
&+ \sum_{i>j}\sum_{\substack{\beta\neq\alpha:\\ \beta\in\mathcal{I}_i\cap\mathcal{I}_j}} \left(\begin{cases} -\frac{m'_\beta}{(M_i)^2 M_j} & \text{if } \alpha\in\mathcal{I}_i \\ -\frac{m'_\beta}{M_i (M_j)^2} & \text{if } \alpha\in\mathcal{I}_j \\ \text{1st} + \text{2nd} & \text{if } \alpha\in\mathcal{I}_i\cap\mathcal{I}_j \end{cases}\right) \\
&\times 2c_i c_j \mathrm{Cov}_\psi\left(\hat{P}_i, \hat{P}_j\right).
\end{aligned} \tag{10}$$

To avoid constrained optimization with m'_α, we use auxiliary variables p_α that express m'_α as

$$m'_\alpha = \frac{e^{p_\alpha}}{\sum_\beta e^{p_\beta}} \tag{11}$$

to introduce the $\sum_\alpha m'_\alpha = 1$ and $m'_\alpha > 0$ conditions. This is known as the softmax function often used in machine learning techniques. Derivatives $\partial \mathrm{Var}\left(\bar{H}\right)/\partial p_\beta$ only require additional terms

$$\frac{\partial m'_\alpha}{\partial p_\beta} = \begin{cases} m'_\beta\left(1 - m'_\beta\right) & \alpha = \beta \\ -m'_\alpha m'_\beta & \alpha \neq \beta \end{cases} \tag{12}$$

for completing a chain-rule expression with Eq. (10).

SUPPLEMENTARY NOTE 4. MEASUREMENT OPTIMALITY IN OVERLAPPING SCHEMES

Here we present an example where an arbitrary choice of numbers of measurements in the measurement allocation (MA) approach results in a higher estimator variance than the

estimator variance for the non-overlapping sorted insertion (SI) method. This may seem counterintuitive at first because MA measures a Pauli product in all possible groups and this should be more efficient compared to measuring the Pauli product only in a single group that takes place in the SI method. Yet, one needs to keep in mind that the MA procedure by choosing the number of measurements for each shareable Pauli product is effectively modifying the coefficient with which this Paul enters the measurable group. This modification affects quantum variance for the group. For optimality, the number of times the group is measured needs to be related to the group variance by Eq. (9) of the main text which is not generally the case for an arbitrary measurement allocation.

Applying the MA and SI methods to the model Hamiltonian

$$\hat{H} = -0.612\hat{x}_1\hat{x}_2 - 2.183\hat{z}_1 - 1.35\hat{z}_1\hat{z}_2, \tag{13}$$

we have the following fragments for the SI approach

$$\hat{A}_1^{(\mathrm{SI})} = -2.183\hat{z}_1 - 1.35\hat{z}_1\hat{z}_2, \tag{14}$$

$$\hat{A}_2^{(\mathrm{SI})} = -0.612\hat{x}_1\hat{x}_2, \tag{15}$$

and their MA counterparts

$$\hat{A}_1^{(\mathrm{MA})} = -2.183\hat{z}_1 - 1.35\frac{m_1}{m_1+m_2}\hat{z}_1\hat{z}_2, \tag{16}$$

$$\hat{A}_2^{(\mathrm{MA})} = -0.612\hat{x}_1\hat{x}_2 - 1.35\frac{m_2}{m_1+m_2}\hat{z}_1\hat{z}_2. \tag{17}$$

To compare the two approaches, we use the same wavefunction

$$|\psi\rangle = (-0.027 - 0.003i)\,|00\rangle + (0.406 + 0.427i)\,|01\rangle$$
$$+(0.614 + 0.042i)\,|10\rangle + (-0.342 + 0.396i)\,|11\rangle\,,$$

where $|ij\rangle$ is the computational basis, and measurement allocation, $m_1 = 0.621$, $m_2 = 0.379$. The resulting estimator variances for MA and SI methods are 6.43 and 6.40 respectively. The MA scheme achieves higher estimator variances because for the $\hat{A}_\alpha^{(\mathrm{MA})}$ fragments the optimal m_α in Eq. (9) of the main text are different than the chosen m_α that gave rise to coefficients of the sharable $\hat{z}_1\hat{z}_2$ products.

SUPPLEMENTARY NOTE 5. RELATION BETWEEN HAMILTONIAN ESTIMATOR VARIANCES AND ENERGY ERRORS

The results presented in Tables 1 and 3 - 5 of the main text are given in terms of variances of Hamiltonian estimators. The central limit theorem (CLT) guarantees that the distribution of the estimator values obtained from measurements converges to a normal distribution centred at the Hamiltonian expectation value and with the variance that is equal to that of the Hamiltonian estimator. This connects the obtained variances of Hamiltonian estimators with probable errors in energy that these estimators provide. Here, we numerically verify this connection through quantum measurement simulations on some representative measurable fragments.

Given some operator, $\hat{O} = \sum_i c_i \hat{P}_i$, we simulate $M_\mathrm{c} = 1000$ quantum measurements of $\hat{O}$ with a wavefunction. From these measurements, we obtain an estimator value, $\bar{O}$:

$$\bar{O} = \frac{1}{M_\mathrm{c}} \sum_{i=1}^{M_\mathrm{c}} O_i, \tag{18}$$

where O_i is the result of the ith measurement of $\hat{O}$. We repeat this process $M_\mathrm{s} = 10,000$ times, each time obtaining an estimator value, $\bar{O}_k$, for the kth set of M_c measurements. The distribution of $\bar{O}_k$'s should be similar to a normal distribution with $\mathrm{SampleVar}\left(\bar{O}\right) \approx \mathrm{Var}_\psi\left(\hat{O}\right)/M_\mathrm{c}$, where

$$\mathrm{Mean}\left(\bar{O}\right) = \frac{1}{M_\mathrm{s}} \sum_{k=1}^{M_\mathrm{s}} \bar{O}_k \tag{19}$$

$$\mathrm{SampleVar}\left(\bar{O}\right) = \frac{1}{M_\mathrm{s}} \sum_{k=1}^{M_\mathrm{s}} (\bar{O}_k - \mathrm{Mean}\left(\bar{O}\right))^2 \tag{20}$$

$$\mathrm{Var}_\psi\left(\hat{O}\right) = \langle \hat{O}^2 \rangle - \langle \hat{O} \rangle^2. \tag{21}$$

Table 1 presents a comparison of $\mathrm{SampleVar}\left(\bar{O}\right)$ obtained from simulations and the expected variance, $\mathrm{Var}_\psi\left(\hat{O}\right)/M_\mathrm{c}$, for some operators occurring in energy measurements for H_2 and LiH. Exact ground eigen-states of corresponding systems were used for all measurement simulations. The results show that the distribution of estimator values obtained from measurements are in line with the calculated theoretical variances.

Additionally, to validate the reliability of our variance estimate with multiple fragments within the coefficient splitting framework, we assess an energy estimator of the full H_2

Hamiltonian. This Hamiltonian can be split into the following overlapping fragments:

$$\hat{H} = \hat{O}^{(1)} + \hat{O}^{(2)} \tag{22}$$

$$\begin{aligned}\hat{O}^{(1)} &= -0.328 + 0.137\hat{z}_0 + 0.137\hat{z}_0\hat{z}_1 - 0.130\hat{z}_2 \\ &\quad -0.130\hat{z}_1\hat{z}_2\hat{z}_3 + \hat{z}_1 + \hat{z}_0\hat{z}_2 + \hat{z}_0\hat{z}_1\hat{z}_2\hat{z}_3 \\ &\quad +\hat{z}_0\hat{z}_1\hat{z}_2 + \hat{z}_0\hat{z}_2\hat{z}_3 + \hat{z}_1\hat{z}_3\end{aligned} \tag{23}$$

$$\begin{aligned}\hat{O}^{(2)} &= -0.843\hat{z}_1 + 0.049\hat{y}_0\hat{z}_1\hat{y}_2\hat{z}_3 + 0.049\hat{x}_0\hat{z}_1\hat{x}_2 \\ &\quad +0.049\hat{x}_0\hat{z}_1\hat{x}_2\hat{z}_3 + 0.049\hat{y}_0\hat{z}_1\hat{y}_2 - 0.894\hat{z}_0\hat{z}_2 \\ &\quad -0.845\hat{z}_0\hat{z}_1\hat{z}_2\hat{z}_3 - 0.845\hat{z}_0\hat{z}_1\hat{z}_2 - 0.894\hat{z}_0\hat{z}_2\hat{z}_3 \\ &\quad -0.837\hat{z}_1\hat{z}_3.\end{aligned} \tag{24}$$

This splitting is different from those used in Tables 1 and 3 - 5 of the main text for H_2, and it is taken here to provide a small illustrative example. Note that $\{\hat{z}_1, \hat{z}_0\hat{z}_2, \hat{z}_0\hat{z}_1\hat{z}_2\hat{z}_3, \hat{z}_0\hat{z}_1\hat{z}_2, \hat{z}_0\hat{z}_2\hat{z}_3, \hat{z}_1\hat{z}_3\}$ are the Pauli products common for both fragments. According to Eq. (7) of the main text, the expected variance of $\bar{H}$ is

$$\mathrm{Var}\left(\bar{H}\right) = \frac{1}{M}\left(\frac{1}{m_1'}\mathrm{Var}_\psi\left(\hat{O}^{(1)}\right) + \frac{1}{m_2'}\mathrm{Var}_\psi\left(\hat{O}^{(2)}\right)\right) \tag{25}$$

where $M = 2000$ is the total number of measurement, and $m_1' = m_2' = 1/2$ are proportions of measurement allocated for corresponding fragments. In Fig. 1, the histogram shows that $\bar{H}$ is normally distributed, in line with CLT, and with variances consistent with Eq. (7) of the main text and Eq. (25).

Supplementary Table 1. Comparison of variances of operator estimators through simulation and expected theoretical variance. All simulations and calculations are done with respect to the ground eigen-state of the corresponding system.

Systems	Operator $(\hat{O})$	SampleVar $(\bar{O})$	$\mathrm{Var}_\psi\left(\hat{O}\right)/M_c$
H_2	$\hat{y}_0\hat{z}_1\hat{y}_2\hat{z}_3$	8.75×10^{-4}	8.81×10^{-4}
H_2	$0.137\hat{z}_0 + 0.137\hat{z}_0\hat{z}_1 - 0.130\hat{z}_2 - 0.130\hat{z}_1\hat{z}_2\hat{z}_3$	3.46×10^{-5}	3.41×10^{-5}
LiH	$\hat{y}_0\hat{x}_1\hat{y}_2$	9.88×10^{-4}	1.00×10^{-3}
LiH	$\hat{z}_0 + \hat{z}_0\hat{z}_1 + \hat{z}_2 + \hat{z}_1\hat{z}_2\hat{z}_3$	2.61×10^{-4}	2.63×10^{-4}

[1] O. Crawford, B. v. Straaten, D. Wang, T. Parks, E. Campbell, and S. Brierley, Efficient quantum measurement of Pauli operators in the presence of finite sampling error, Quantum **5**, 385 (2021).

Supplementary Figure 1. Histogram of estimator results for measurement simulation of the H_2 Hamiltonian in a coefficient split grouping (see Eqs. (23) and (24)), with respect to a random complex wavefunction. Data were collected from $M_s = 10,000$ experiments, and within each experiment, $= 1000$ measurements were performed on each fragment to obtain an estimator value. The simulated distribution has sample variance $\text{SampleVar}\left(\bar{H}\right) = 6.090 \times 10^{-3}$ where the theoretical variance is $\text{Var}\left(\bar{H}\right) = 6.130 \times 10^{-3}$.

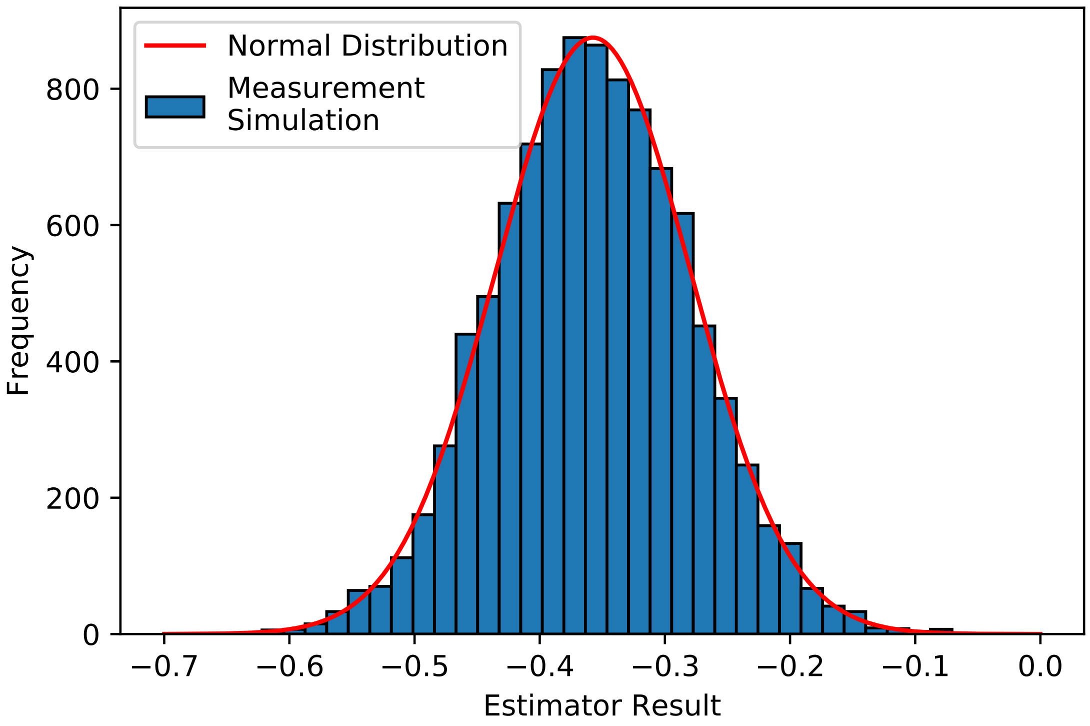

Supplement: Supplementary file 1 — Deterministic improvements of quantum measurements with grouping of compatible operators, non-local transformations, and covariance estimates [file 41534_2023_683_MOESM1_ESM.pdf]
